# Service Use Objectives among Older Adult Day Care Clients with Disability in Japan

**DOI:** 10.3390/nursrep11030058

**Published:** 2021-08-02

**Authors:** Takashi Naruse, Noriko Yamamoto-Mitani

**Affiliations:** 1 Global Nursing Research Center, Graduate School of Medicine, The University of Tokyo, Tokyo 113-0033, Japan; 2 Department of Gerontological Home Care and Long-Term Care Nursing/Palliative Care Nursing, Graduate School of Medicine, The University of Tokyo, Tokyo 113-0033, Japan; noriko-tky@g.ecc.u-tokyo.ac.jp

**Keywords:** adult day care, aged care, quality assurance, long-term care, outcome measurement, service needs

## Abstract

Quality assurance in long-term care settings requires outcome evaluation reflecting client-specific needs of service use. This study aimed to explore the clients’ needs of adult day care (ADC). Data of 360 clients from 11 ADC agencies in Japan were analyzed. Clients’ needs for ADC use were evaluated by their respective ADC staff using 17 items of four domains: “social participation,” “hygiene and health,” “exercise and eating habits,” and “family support.” The prevalence of four domain needs was calculated and the relationship between physical independency and the presence of needs in the four domains was examined by the chi-squared test. A total of 291 (80.8%) clients had one or more needs while 69 (19.2%) clients had none. The social participation need was most prevalent (270, 75.0%) and 249 clients (69.1%) had combination needs, including social participation, along with another domain. “Feeling like revisiting the ADC” was the most common need (60.6%); it was more frequently needed by those with a higher level of independence (*p* = 0.003). The study findings suggest that an outcome measure relevant to social participation can be considered as the most common benefits of ADC use. However, ADCs with clients who are more dependent should consider hygiene, nursing, and family support needs.

## 1. Introduction

Quality assurance in long-term care (LTC) settings supports older persons to continue living safely and comfortably [1]. In the community-based integrated care system [2], older persons receive LTC in various contexts, including hospitals, residential facilities, and their own homes, depending on health status or social needs. Reports have found an increase in the proportion of LTC recipients living at home; this is a major consequence of rapidly aging populations in OECD countries, including Japan [3]. Hence, these countries have witnessed a growing interest in adult day care (ADC) services/centers/agencies for the older population with disabilities. ADC is a generic term for aged care; it comprises building-based services that offer a wide variety of programs and amenities for older people [4]. In Japan, ADC is a type of LTC service. In April 2018, with 1.13 million ADC clients, ADC was the most popular type of LTC service among the Japanese disabled population [5]. ADC clients’ intervention-related benefits include improved physical, mental, and social function, exposure to comprehensive care, improved well-being, and alleviation of family caregivers’ burdens [4,6,7].

The Japanese clients’ experiences during Adult Day Care service include the use of the J-AdaCa Tool which has been developed to assess the richness of clients’ experiences regarding their ADC service use [8]. It comprises four factors regarding experience of ADC use: “social participation,” “hygiene and health,” “exercise and eating habits,” and “family support”. The nature of ADC with four components was in line with an international review that reported four general aims of ADC: providing social and preventive services, supporting clients’ continued independence, supporting clients’ health and daily living needs, and enabling family caregivers to take breaks from daily care and/or continue with their employment [4]. While quality assurance for clients of ADC services is important, there was no consensus about the appropriate outcome index for measurement. Owing to ADC clients’ and staff’s heterogeneity, it is difficult to objectively demonstrate the benefits of ADC programs and interventions [9]. The four aspects of service experience could differentially benefit clients. Thus, the best benefit outcome measure for each client should depend on their needs. In rehabilitation-specific ADCs, the degree of clients’ ADL impairments was reported to be associated with their objective on ADC [10].

This study analyzed data to explore the clients’ needs of ADC use. In the case of common needs of ADC use, its sequence could be a possible benefit index of ADC services. To add a description of the nature of service needs according to the clients’ background, the association between service needs and physical independency was examined.

## 2. Materials and Methods

This study analyzed data obtained from the ADC chart in an earlier study that developed the J-AdaCa tool [8]. The survey was conducted from September 2019 to March 2020 in Tokyo, Japan. Study instructions, consent forms, and questionnaires were mailed to the administrators of the 15 aforementioned agencies. If administrators answered the consent form, it was implied that the ADC agency agreed to collaborate. Regarding the questionnaire application, administrators chose a typical service day (chosen based on their own administrative settings) for the administration of the survey in their respective ADC agencies. Administrators explained the purpose and methodology of this study to their respective ADC clients. The inclusion criterion was clients who were using the ADC and its services for three months or longer. There were no exclusion criteria. To ensure participants complied with this criterion, one ADC staff (i.e., either the chief of staff in the respective center or a staff member who had thorough knowledge of clients’ current status) gathered data about clients who visited their agency on the day of the survey application; this data was gathered from chart information and administrators’ subjective assessments. Completed consent forms and questionnaires from ADC administrators/staff were mailed to the researcher.

Since principal demographic data (except for names) were collected from clients’ charts by the ADC staff, these clients were not required to provide a written consent form.

### 2.1. Measures

The examined variables included clients’ demographic information, activities of daily living (ADL), and dementia severity. Demographic information included age, gender, living condition, and frequency and history of ADC service use.

The ADC clients’ ADL was measured by clients’ difficulty in standing up based on a two-point scale: “need assistance” or “independent”.

Dementia severity was measured by two items of the Inter Resident Assessment Instrument (Inter-RAI, home version) from a Japanese guidebook (the original guidebook has seven items) [11]. From the Japanese guidebook, we included items on socially inappropriate behavior and refusing care; these two items were chosen by research collaborators, as they were common problem behaviors among ADC clients (while the others were not recurrent). Given our study aims (to examine problematic behaviors frequency in ADC settings), the four-point scale was modified to: “Each time this client came to the ADC agency,” “Once for every two times this client came to the ADC agency,” “Sometimes, but less than once for every two times this client came to the ADC agency,” and “Not at all when this client came to the ADC agency”.

Clients’ needs for ADC use was measured using the J-AdaCa tool items [8]. The ADC staff was asked about the degree of importance of each need in ADC care; they responded to the 17 items (based on their self-assessments) on a thre-point scale: 1 = not important, 2 = important but not their objective for use ADC, and 3 = important because it is their objective for use ADC. The 17 items were assigned to four “ADC needs domains” by referring to the results of the principle component analysis of J-AdaCa tool. Eight items (e.g., “comfortable mealtime” and “using one’s five senses”) were included in the “social participation” domain. Five items (e.g., “Being advised in the amount ingested”) were in the “hygiene and health” domain, three items (e.g., “Exercising more than enough”) were in the “exercise and eating habits” domain, and two items (e.g., “Family member being away from the care recipient”) were in “family support” domain. If one or more items within each domain were evaluated as “objective for ADC use”, the domain was considered as “presence of needs in this domain of ADC use”.

### 2.2. Data Analysis

Statistical analyses were conducted by SPSS version 24 (IBM Japan, Ltd., Tokyo, Japan). Frequencies and means were calculated on all measures. First, the prevalence of four domain needs were calculated. The presence of combination needs was described by a Venn diagram and the most common domain was identified. Second, the relationship between physical independency and the presence of needs in four domains/17 items was examined by *t*-test or chi-squared test. The level of significance was set at 0.05.

## 3. Results

Among 15 ADC agencies that were interested in participating in this study, we collected data from 11 ADC agencies; further, 360 clients met the inclusion criteria. While the other four agencies were interested in participating in this study, owing to the spread of COVID-19 in Japan [12], the researchers chose to cancel their participation. The cancellation was communicated in February 2020. To avoid confusion among ADC staff and clients, all surveys of these agencies were cancelled.

### 3.1. Characteristics of Participants

Clients had a mean age of 85.4 (standard deviation [SD] = 6.8; range: 55–102) years. In total, 263 clients (73.1%) were women and 105 (29.2%) lived alone. About 80% reported no problem with dementia in the two analyzed behaviors; 80.6% in “socially inappropriate behavior” and 81.4% in “refusing care”. Regarding ADL, 31.7% of the clients needed assistance in toileting, 29.4% when standing up (29.4%), and 13.9% when eating. On an average, clients visited the ADC 2.6 (SD = 2.0: 1–6) times a week and had used it for 4.1 (SD = 3.8: 1–20) years.

### 3.2. The Combination of Four Needs Domains

A total of 291 (80.8%) clients had one or more needs and 69 (76.7%) had none. The social participation need was the most prevalent (270, 75.0%), followed by hygiene and health (229, 63.6%), exercise and eating habits (201, 55.8%), and family support (157, 43.6%).

The presence of the combination of four needs domains and its prevalence is shown in Table 1. Among the 270 clients with social participation needs, 21 (5.8%) of them had social participation only, the other 249 (69.1%) clients had combination needs comprising social participation and other domains. The most prevalent was the segmentation of clients who had all needs domains (31.7%). The second was clients who did not have any needs domain (19.2%), and next to who had social participation, hygiene and health, and exercise and eating habits (16.1%). A total of 47.8% (31.7% and 16.1%) clients needed three domains with/without family support domain.

There was no association between the presence of needs in each domain and physical independency.

### 3.3. Relationship between Need Items in Four Domains and Level of Independence

Among the eight items in the social participation domain, “Feeling like revisiting here” was the most common response for 218 clients (60.6%), followed by “Recognizing one’s own place in society” for 212 clients (58.9%). Both these items were needed more frequently by those with a higher level of independence (*p* = 0.003 and <0.001, respectively). In general, 40–60% clients had needs for all the social participation domain items (Table 2). However, “Recognizing value in helping others” was the least commonly found need, with only 111 respondents (30.8%) reporting this need. Only 14.2% dependent clients had a need (*p* < 0.001).

### 3.4. Characteristics of Those with No Purpose

Additional comparative analyses were conducted to identify the characteristics of those with no needs. None of the variables showed any significant association with the absence of service needs.

## 4. Discussion

The study findings reveal that older adults require professional support in terms of their social participation and/or in terms of hygiene, exercise and eating, and family support. Prior literature revealed that it was difficult to measure service use benefits among ADC clients, due to heterogeneity of their needs [9]. However, as social participation was the specific objective of ADC use for 75% clients, the outcome reflecting better fulfillment of socialization needs can be a common index for quality ADC service. Because the prevalence of clients with social participation needs was over 90% in clients with 291 clients with one or more needs, it is a primary ADC benefit for clients who had any specific objectives.

Fulfilling socialization needs can lead to improved health because social participation is shown to improve clients’ physical and mental health as well as promote positive social relationships [13]. Moreover, prior studies have shown that access to the world through ADC activities improved clients’ psychosocial wellbeing [14]. Additionally, social participation makes older people more stimulated, confident, and content [15]. A recent systematic review about ADC effectiveness also implicated the benefits of socialization for ADC [4]; however, there was little evidence regarding how care providers or the ADC center environment can promote clients’ social participation. Future studies should use the measurement tool of ADC service experiences to identify clients with “high social participation needs” and assess their service experiences. Moreover, a comparison between clients with and without enough socialization experiences will demonstrate the impact of ADC staff/environment on client’s socialization experiences. This will help health providers and administrators understand how their client’s socialization needs can be fulfilled.

When considering the client segmentation explained by combination of needs domains, 47.8% clients needed three domains with/without family support domain. There was a small number of clients who had only one specific needs domain. About a half of ADC clients can accept outcome measurement relevant to hygiene and health, and exercise and eating habits. ADC would be expected to respond to a combination of multiple needs [16] and contribute to comprehensive benefit of each client.

There was no association between the presence of needs in each domain and physi-cal independency but five items in social participation and two items in other domains showed significant different prevalence by clients’ physical dependency.

Among eight needs items in social participation, the more independent the client was, the more likely they needed the specific five contents (i.e., comfortable mealtime, speaking aggressively, recognizing value in helping others, recognizing one’s own place in society, and feeling like revisiting here). As previously discussed, social participation might be a fundamental ADC need among physically independent as well as physically dependent clients, but these two groups may have different kinds of social participation needs. Among independent clients, the most common three needs were “recognizing one’s own place in society (65.4%)”, “feeling like revisiting here (65.0%)” and “comfortable mealtime (50.8%)”. Among the dependent clients, these needs were “feeling like revisiting here (50.0%)”, “recognizing one’s own place in society (43.4%)” and “using one’s five senses (40.6%)”. This difference was considered to indicate the nature of different social participation needs, where independent clients enjoy mealtime; however, dependent clients may receive stimulations based on other people’s behavior or environment around themselves.

“Cleaning oneself” in the hygiene and health domain and “family members being supported” in the family support domain were found to be more common among dependent clients (46.2% and 28.7%, respectively) than in independent clients (38.1% and 18.4%, respectively). Being the results of physical dependency, these may be distinctive ADC needs. When discussing the issue of “cleaning oneself,” we should add an explanation about the unique background of Japanese old people. In particular, Japanese old people take great enjoyment in experiencing the Japanese bath [17]. However, only 23% of housing units in Japan were reported to have easy-straddle bathtubs [18]; thus, it is difficult for physically dependent old people to soak in a bath in their own home. Our results might reflect that, nevertheless, out of physical dependency, Japanese old people prefer to take baths. One national survey showed that over 90% of ADCs provided bathing care [19].

Our current participants included 29.4% dependent persons who could not stand up by themselves. LTC clients are estimated to increase and get more severe in the next three decades in Japan [2]. As there will be more dependent clients in future ADCs, the primary index for ADC service quality should focus on “cleaning oneself” and “family members being supported” to a greater extent. On the other hand, the priority according to social participation, in particular, the five social participation items, could be lower. The primary index should be updated on a timely basis according to the changes in clients’ dependency.

### Implications for ADC Service Quality Assessment

The study findings highlight that to assess the quality of care in ADC agencies and compare ADC agencies, an index relevant to social participation is recommended. However, 25% clients did not have needs regarding social participation. For ADCs with clients who are more dependent, other indexes relevant to hygiene, nursing, and family support should be considered. Because each client tended to have multiple needs from four domains, a case mix for service quality evaluation in ADCs is warranted. Future research must clarify the association between clients’ characteristics and their ADC needs. Any related research may relate to the development of an outcome prediction model based on client characteristics that form the basic development approach for a case mix indicator.

## Figures and Tables

**Table 1:** The combination of four needs domains among the adult day care clients.

Group of Clients with Combination of Needs ^1^					*n*	(%)
Id	SP	HH	EE	FS		
SP	+	−	−	−	21	(5.8)
SP/HH	+	+	−	−	33	(9.2)
SP/EE	+	−	+	−	10	(2.8)
SP/FS	+	−	−	+	7	(1.9)
SP/HH/EE	+	+	+	−	58	(16.1)
SP/HH/FS	+	+	−	+	19	(5.3)
SP/EE/FS	+	−	+	+	8	(2.2)
SP/HH/EE/FS	+	+	+	+	114	(31.7)
HH	−	+	−	−	4	(1.1)
HH/EE	−	+	+	−	0	(0.0)
HH/FS	−	+	−	+	1	(0.3)
HH/EE/FS	−	+	+	+	0	(0.0)
EE	−	−	+	−	8	(2.2)
EE/FS	−	−	+	+	3	(0.8)
FS	−	−	−	+	5	(1.4)
Absent	−	−	−	−	69	(19.2)

^1^ SP: Social participation, HH: Hygiene and health, EE: Exercise and eating habits, and FS: Family support.

**Table 2:** Relationship between needs items in four domains and level of independence in standing up.

Need Domain ^1^	Need Items	Total		Independent		Dependent		
		*n ^2^*	(%)	*n ^2^*	(%)	*n ^2^*	(%)	*p* ^3^
SP	Comfortable mealtime	163	(45.3)	129	(50.8)	34	(32.1)	0.001
	Using one’s five senses	167	(46.4)	124	(48.8)	43	(40.6)	0.165
	Speaking aggressively	148	(41.1)	115	(45.3)	33	(31.1)	0.014
	Classifying ideas	144	(40.0)	108	(42.5)	36	(34.0)	0.127
	Recognizing value in helping others	111	(30.8)	96	(37.8)	15	(14.2)	<0.001
	Recognizing one’s own place in society	212	(58.9)	166	(65.4)	46	(43.4)	<0.001
	Feeling like revisiting here	218	(60.6)	165	(65.0)	53	(50.0)	0.009
SP and EE	Eating more than usual	143	(39.7)	106	(41.7)	37	(34.9)	0.237
EE	Exercising more than enough	190	(52.8)	136	(53.5)	54	(50.9)	0.728
	Exercising unconsciously	179	(49.7)	132	(52.0)	47	(44.3)	0.295
HH	Being advised of the amount ingested	76	(21.1)	53	(20.9)	23	(21.7)	0.887
	Cleaning oneself	137	(38.1)	88	(34.6)	49	(46.2)	0.037
	Receiving assessment	118	(32.8)	86	(33.9)	32	(30.2)	0.621
	Receiving treatment	82	(22.8)	55	(21.7)	27	(25.5)	0.491
	Receiving explanations about one’s health conditions	79	(21.9)	52	(20.5)	27	(25.5)	0.327
FS	Family member being away from the care recipient	131	(52.4)	82	(50.3)	49	(56.3)	0.241
	Family members being supported	55	(22.0)	30	(18.4)	25	(28.7)	0.020

^1^ SP: Social participation, ADC: Adult day care, HH: Hygiene and health, EE: Exercise and eating habits, and FS: Family support. ^2^ *n*: number of clients who had need for each item within Adult day care. ^3^ *p*-value for Chi-squared test.
